# Identifying high-risk combinations of metformin during COVID-19

**DOI:** 10.1371/journal.pone.0343979

**Published:** 2026-03-04

**Authors:** Jelena Dimnjaković, Tamara Buble, Tamara Poljičanin, Hana Brborović, Emanuel Brađašević, Ognjen Brborović

**Affiliations:** 1 Department for Biostatistics, Division for Health Informatics and Biostatistics, Croatian Institute of Public Health, Zagreb, Croatia; 2 Health center "Dom zdravlja Zagreb zapad", Zagreb, Croatia; 3 Department of Environmental and Occupational Health and Sports Medicine, School of Medicine, University of Zagreb, Zagreb, Croatia; 4 Department of Social Medicine and Organization of Health Care, School of Medicine, University of Zagreb, Zagreb, Croatia; Shuguang Hospital, CHINA

## Abstract

**Background:**

There is a lack of research addressing associations of antidiabetic drug combinations with COVID-19 deaths. We examined whether adding common second-line agents to metformin was associated with COVID-19 mortality risk to inform clinical decision-making when escalating diabetes treatment.

**Methods:**

This is a nationwide retrospective analysis covering the years 2020 and 2021. Data from the National Diabetes Registry (CroDiab) were linked to primary healthcare data, Causes of Death Registry data, and the SARS-CoV-2 vaccination database. Multivariate logistic regression models were developed for each of the combinations to compare the combination with metformin monotherapy. To address confounders, inverse probability of treatment weighting (IPTW) analysis as well as analysis with stabilized weights was performed.

**Results:**

Of 141014 analyzed patients, 1268 (0.90%) died of COVID-19 in 2 years. Weighted results of the drug combinations that showed statistically significant associations to COVID-19 death in comparison to metformin alone were metformin+DPP-4 inhibitor (OR 1.182, 95% CI 1.016–1.376), metformin+sulfonylurea (OR 1.195, 95% CI 1.015–1.406), and metformin+GLP-1 agonist (OR 2.992, 95% CI 2.117–4.229).

**Conclusions:**

Some combinations of metformin with second-line antidiabetic drugs might require caution in the context of chronic diabetes mellitus type 2 therapy and COVID-19 related deaths. Findings should be interpreted as hypothesis-generating signals from real-world data rather than evidence of causal drug effects. Further research is needed, especially for metformin+GLP-1 agonist, as well as head-to-head comparisons of combinations therapies.

## Introduction

It is estimated that over 500 million people aged 20–79 have diabetes mellitus and every year, 6.7 million people die due to diabetes [1]. During the COVID-19 pandemic, people with diabetes mellitus faced a significantly higher risk of severe outcomes and mortality compared to individuals without diabetes [2]. SARS-CoV-2, the virus that causes COVID-19, caused pandemics in the years 2020–2023 is still present worldwide causing cumulatively up to July 2024 7.1 million deaths [3,4].

At the start of the COVID-19 pandemic, there were concerns that some antidiabetic drugs might worsen outcomes, although their anti-inflammatory properties suggested possible benefits [5–9]. Many meta-analyses of observational studies evaluated associations between major antidiabetic drug classes and COVID-19 outcomes. Results of those meta-analyses consistently show that metformin decreases COVID-19 mortality risk by 22%−46% [10–20], while insulin increases it 1.38–2.59 times [10,15,17,19,21,22]. Meta-analyses mostly show that SGLT-2 inhibitors reduce mortality risk by 18%−40% [10,15,19,23]. Also, GLP-1 agonists mostly reduce mortality risk by 9%−49% [10,11,15,19,24]. Findings for sulfonylureas are mixed: some meta-analyses suggest a 7%−20% mortality decrease [12,17], while others show non-significant associations [10,15,19]. Similarly, results for DPP-4 inhibitors range from a 12%−42% risk reduction [25–27] to non-significant findings [10–12,15,17,28–30].

However, there is lack of studies addressing association of antidiabetic drug combinations and COVID-19 outcomes. This gap is critical given that long-term monotherapy of diabetes mellitus is often ineffective in achiving HbA1c goals [31,32]. A randomized trial demonstrated that with metformin monotherapy, 21% of patients lost glycemic control after five years [33]. Consequently, many patients require at least two antidiabetic drugs for adequate glycemic management.

Metformin is the most used antidiabetic drug and is usually first choice drug in treating diabetes mellitus type 2. It is the most prescribed first-line antidiabetic drug [34]. It acts primarily by reducing peripheral insulin resistance and inhibiting hepatic gluconeogenesis [35,36]. Its molecular target, the adenosine monophosphate-activated protein kinase (AMPK), plays a key role in mitochondrial homeostasis and energy regulation. Activation of AMPK triggers several downstream pathways thought to mediate the therapeutic effects of metformin, although its full mechanism remains incompletely understood [35]. Metformin typically lowers HbA1c% by about 0.9 percentage points, is weight-neutral or modestly weight-reducing and does not cause hypoglycemia [34,37,38]. It may also bring cardiovascular (CV) protection through AMPK-dependent anti-atherogenic and anti-thrombotic effects, with potential benefits in myocardial injury, ischemia, and diabetic cardiomyopathy [34,39].

Our study therefore compared metformin-based combination therapies to metformin monotherapy, reflecting the clinical decision point when escalation beyond first-line therapy becomes necessary. The study can help clinicians in making decisions which antidiabetic to add to metformin once monotherapy is not enough.

## Materials and methods

The study was a retrospective data analysis covering the period from Jan 1st 2020 to Dec 31st 2021 Croatian National Diabetes Registry (CroDiab) was the source of data.

CroDiab contains individual longitudinal data on patients with diabetes mellitus. Several sources are being used to feed CroDiab with data via the National Public Health Information System of Croatia and the Central Health Information System of the Republic of Croatia: clinical laboratories, primary health care providers, and hospitals. For our study, CroDiab was linked to a database containing SARS-CoV-2 test results, the National Vaccination Database (eVac), and the National Causes of Death Registry using a common personal identifier by a designated team member [40]. The resulting data export was anonymized. This anonymized dataset was transferred to another team member for statistical analyses; all other team members received access to aggregated results only.

Conditions for defining a person with diabetes mellitus type 2, COVID-19 death, anti-diabetic drug intake, and a comorbidity were as follows. A person is classified as a person with diabetes mellitus if at least one of the following conditions are met: (1) at least one hospital report with diabetes mellitus diagnosis was found in the system, (2) if the person visited their primary healthcare provider at least twice in period of study and ICD-10 diagnosis of E11 was recorded during the visits, (3) if the person was prescribed at least two prescriptions with diagnosis E11 or if the prescriptions had Anatomical Therapeutic Chemical Classification (ATC) codes A10 excluding code A10BA, (4) if person's primary healthcare provider reported the person as diabetes mellitus patient via the National Public Health Information System plus the person visited her primary healthcare provider at least once and ICD-10 diagnosis of E11 was recorded during the visit or the person was prescribed at least one prescription with diagnosis E11 or if the prescription had ATC codes A10 excluding code A10BA.

Anti-diabetic drug intake was defined as a prescription that was prescribed at least two times during eight months before the SARS-CoV-2 or COVID-19 outcome. If the person experienced none of the outcomes, therapy was defined if a prescription was picked up at least once eight months before the patient visited her primary healthcare provider with a diagnosis of diabetes mellitus recorded in the system during that visit.

COVID-19 death outcome was defined as death with COVID-19 listed as the primary source of death per the National Causes of Death Registry. The diagnosis of COVID-19 was determined per the World Health Organization International Statistical Classification of Diseases and Related Health Problems, 10th revision (ICD-10), code U07. All COVID-19 diagnoses were laboratory-confirmed by Polymerase Chain Reaction (PCR) test.

Individual comorbidities were identified if their ICD-10 codes were recorded at least twice in the system from Jan 1st 2018 onwards. ICD-10 codes searched for were as follows: malignant neoplasms (C00-C97); hypertensive diseases (I10-I15); ischemic heart diseases (I20-I25); cerebrovascular diseases (I60-I69); diseases of the circulatory system excluding hypertension (I00-I09 and I20-I99); chronic lower respiratory diseases (J40-J47); other chronic obstructive pulmonary disease (J44); cardiomyopathy (I48).

Inclusion criteria for data analysis were presence of type 2 diabetes mellitus defined as per CroDiab definition described above, age ≥ 18 years and use of any antidiabetic drug as described above. Patients who did have diagnosis of diabetes mellitus type 2 but no information on antidiabetic drug use was available, were excluded from analysis [40].

No sample size calculation was performed, as this retrospective analysis included the complete eligible population from the national registry.

### Logistic regression models

Multivariate models were developed. For each model a new variable was created – e.g., if we wanted to compare metformin+DPP-4 inhibitor vs metformin alone then variable called “metformin+DPP4 vs metformin only” was created. It had 2 categories: category 1 was metformin alone and category 2 combination metformin+DPP-4 inhibitor. That is how we assured that combination will be compared against metformin in regression model. Regression model was performed on patients taking either metformin alone or metformin+DPP-4 inhibitor and no other antidiabetic drugs. Equation of the general structure of the logistic regression was:

logit(P(COVID-19 death)) = β₀ + β₁(drug combination) + ∑βᵢ(covariates) where

P = probability of the outcome, β₀ = the intercept, β₁ = the coefficient for the drug combination variable, βᵢ = the regression coefficients for each covariate.

Clinically relevant covariates identified through prior research were included in all regression models: age, diabetes duration, sex, ACEI use, ARB use, SARS-CoV-2 vaccination dose 1, dose 2, booster dose, comorbidities: neoplasm, arterial hypertension, ischemic heart disease, cardiomyopathy, cerebrovascular diseases, circulatory diseases except hypertension, chronic lower respiratory diseases, other chronic obstructive lung diseases, chronic kidney disease [41].

The diabetes duration variable was categorized as “diagnosed 2013 or earlier” versus “2014 onward” to account for systemic reporting changes in national data collection practices. HbA1c and BMI were excluded from multivariate models due to insufficient completeness across the study population.

In the multivariate models, Odds ratios (OR) and 95% CI were determined.

Full models as well as numbers of patients and numbers of death outcomes in each group are available in S1-S15 Tables, in Supplemental file.

### Inverse probability of treatment weighting (IPTW)

Confounding by selection may arise in real-world studies when there are differences in the patients’ characteristics with different treatments being compared [42]. Thus, a sensitivity analysis was performed by using inverse probability treatment weighting (IPTW), assigning to each patient the inverse of the probability of receiving treatment, to control for these differences. The weight average must be approximately equal to 1 to consider the well-behaved weights that lead to a small variance of the effect estimate [42,43]. Since in our analysis mean of IPTWs was not near 1 for any of the analyzed combinations, analysis was adjusted also for stabilized weights [42,44].

For balance diagnostics, standardized mean differences (SMDs) were calculated prior to and after weighing [45]. SMD < 0.1 was considered well balanced [46].

For combinations of metformin+DPP-4 inhibitor, metformin+SGLT-2 inhibitor, metformin+sulfonylurea and metformin+pioglitazone, following the weighing with standardized weights, SMDs for all 18 variables were <0.1 indicating balance. For combination metformin+GLP-1 agonist this was true for 16 of 18 variables. After weighing, variables arterial hypertension and patient age had SMDs + 0.22 and −0.19, respectively. Although IPTW-weighing did not fully reduce imbalances below the pre-specified SMD threshold of 0.10 for a 2 variables in analyzing 1 combination, we considered these balance diagnostics to be indicative of an adequate propensity score model [47].

### Ethics

The Croatian Institute of Public Health Ethical Committee (No. 381-15-21-3) and the University of Zagreb School of Medicine Ethical Committee (No. 641–01/22–02/01) approved the study. The need for informed consent was waived by The Croatian Institute of Public Health Ethical Committee. The study has been performed in accordance with the Declaration of Helsinki.

## Results

In the CroDiab database, there were 231796 patients with diabetes mellitus type 2 matching the inclusion criteria. After removing patients not taking studies drugs or taking them in combinations other than set by the study plan, the study population consisted of 141014 patients with diabetes mellitus type 2.

**Fig 1:**
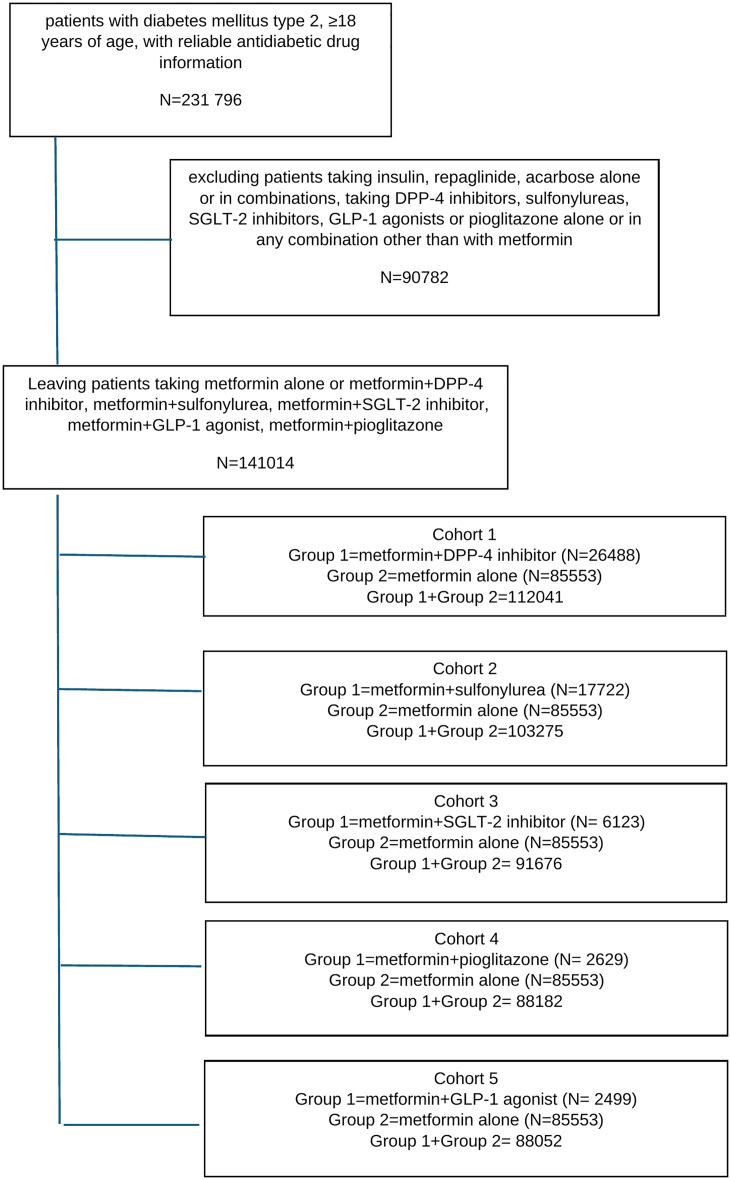
Study flowchart.

85553 were users of metformin alone, 26488 users of metformin+DPP-4 inhibitor, 17722 of metformin+sulfonylurea, 6123 of metformin+SGLT-2 inhibitor, 2629 of metformin+pioglitazone, 2499 of metformin+GLP-1 agonist. The study flowchart is shown in Fig 1.

Number of COVID-19 deaths in studied population was 1268 (0.90%).

Cohorts’ characteristics are provided in Table 1. Between-group differences and SMDs prior and after weighting are provided in S1-S15 Tables, in Supplemental material.

**Table 1:** Cohorts’ characteristics.

Variable	metformin alone (N = 85553)		metformin+ DPP-4 inhibitor (N = 26488)		metformin+ sulfonylurea (N = 17722)		metformin+SGLT-2 inhibitor (N = 6123)		metformin+ pioglitazone (N = 2629)		metformin+GLP-1 agonist (N = 2499)	
Age, mean±SD	65.84 ± 11.80		65.06 ± 10.45		70.55 ± 10.32		60.46 ± 10.28		62.82 ± 10.62		60.05 ± 10.10	
	N	%	N	%	N	%	N	%	N	%	N	%
diabetes duration (>7 years)	21758	25.40	11563	43.70	11480	64.80	2141	35.00	1073	40.80	1277	51.10
sex (female)	44051	51.50	12003	45.30	8725	49.20	1926	31.50	1281	48.70	1405	56.20
ACEI	43356	50.70	13553	51.20	9777	55.20	3223	52.60	1410	53.60	1263	50.50
ARB	2754	3.20	824	3.10	624	3.50	211	3.40	82	3.10	112	4.50
SARS-CoV-2 vaccination, min. one dose	64314	75.20	20056	75.70	12231	69.00	4921	80.40	2021	76.90	2030	81.20
SARS-CoV-2 positivity	11752	13.70	3873	14.60	2488	14.00	983	16.10	370	14.10	391	15.60
COVID-19 hospitalization	2885	3.40	977	3.70	985	5.60	181	3.00	87	3.30	83	3.30
COVID-19 death	692	0.80	233	0.90	273	1.50	33	0.50	15	0.60	22	0.90
cancer	7648	8.90	2300	8.70	1708	9.60	394	6.40	170	6.50	172	6.90
cerebrovascular diseases	4652	5.40	1528	5.80	1270	7.20	311	5.10	147	5.60	151	6.00
arterial hypertension	66717	78.00	20456	77.20	14586	82.30	4670	76.30	2047	77.90	2126	85.10
ischemic heart disease	10327	12.10	3342	12.60	2504	14.10	1341	21.90	197	7.50	350	14.00
cardiomyopathy	3918	4.60	1124	4.20	1155	6.50	382	6.20	76	2.90	134	5.40
circ. diseases other than hypertension	29829	34.90	8981	33.90	6873	38.80	2464	40.20	721	27.40	970	38.80
at least 1 cardiovascular disease	70746	82.70	21674	81.80	15379	86.80	5055	82.60	2137	81.30	2217	88.70
all cardiovascular diseases	998	1.20	342	1.30	319	1.80	143	2.30	26	1.00	40	1.60
lower respiratory tract chronic diseases	8488	9.90	2416	9.10	1719	9.70	549	9.00	239	9.10	353	14.10
other obstructive lung diseases	4335	5.10	1258	4.70	965	5.40	271	4.40	125	4.80	174	7.00
lower respiratory chronic and obstr. together	12823	15.00	3674	13.80	2684	15.10	820	13.40	364	13.90	527	21.10
chronic kidney disease	1062	1.20	399	1.50	333	1.90	70	1.10	30	1.10	54	2.20

SD = standard deviation; DPP-4 = Dipeptidyl peptidase 4, SGLT-2 = Sodium-glucose co-transporter 2, GLP-1 = Glucagon-like peptide-1, ACEI = Angiotensin-converting enzyme inhibitors, ARB = Angiotensin receptor blockers, COVID-19 = coronavirus disease 19, SARS-CoV-2 = Severe acute respiratory syndrome coronavirus 2.

Numbers of events are shown in Table 2.

**Table 2:** Numbers of COVID-19 deaths.

comparator	comparator events		metformin alone events	
	N	%	N	%
metformin+DPP-4 inhibitor	233	0.90	692	0.80
metformin+sulfonylurea	273	1.50	692	0.80
metformin+SGLT-2 inhibitor	33	0.50	692	0.80
metformin+pioglitazone	15	0.60	692	0.80
metformin+GLP-1 agonist	22	0.90	692	0.80

DPP-4 = Dipeptidyl peptidase 4, SGLT-2 = Sodium-glucose co-transporter 2, GLP-1 = Glucagon-like peptide-1.

**Fig 2:**
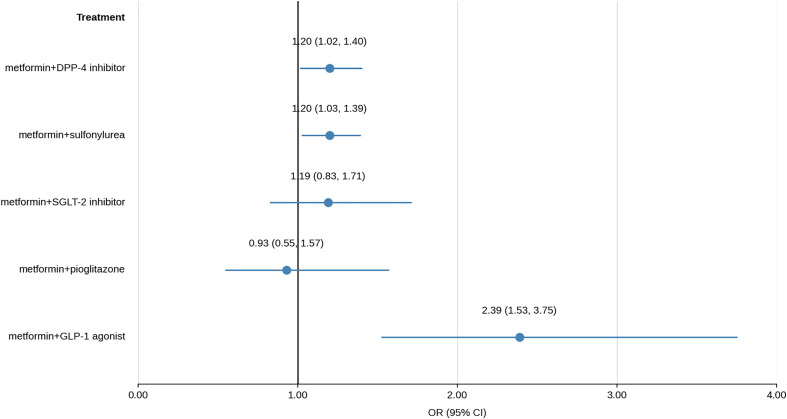
Results of multivariate logistic models prior weighing.

**Fig 3:**
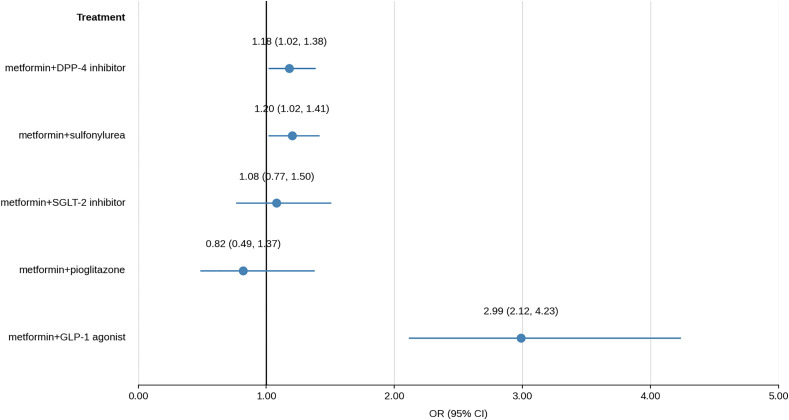
Results of multivariate logistic regression models after weighing.

Results of multivariate logistic models, prior and after weighing, are shown in Fig 2 and Fig 3, respectively.

Patients who are taking metformin+DPP-4 inhibitors, in comparison to patients taking metformin alone, have 1.18 times greater odds of COVID-19 death. Patients taking metformin+sulfonylurea, in comparison to metformin alone, have 1.20 times greater odds, while patients taking metformin+GLP-1 agonist have 2.99 times greater odds of COVID-19 death in comparison to patients taking metformin alone. Results prior and after weighing are similar.

## Discussion

There is a lack of study of antidiabetic drug combinations and their associations with COVID-19 deaths. On the other hand, there are lot of published studies on associations of antidiabetic drug classes and COVID-19 outcomes where risks of users vs non-users are compared.

Meta-analyses of these studies generally show that metformin, SGLT-2 inhibitors and GLP-1 agonist, when comparing users vs non-users, show either protective association against COVID-19 death or at least a neutral one [10]. Sulfonylurea users vs non-users mostly show increase in COVID-19 death risks. DPP-4 inhibitors and pioglitazone show mixed results [10,26].

Metformin, SGLT-2 inhibitors and GLP-1 agonists generally have good cardiovascular (CV) safety profile [48–51]. Sulfonylurea on the other hand does not [52]. For DPP-4 inhibitors and pioglitazone there are mixed data on CV safety profile [53,54].

Thus, perhaps it can be postulated that drugs that generally have favorable CV safety profiles are not associated with increased risks of COVID-19 deaths while drugs which may represent CV strain also show association to increased risk of COVID-19 death. This is in line with COVID-19 raising CV risks itself [55].

In our study, adding sulfonylurea to metformin led to increased COVID-19 death risk in comparison to metformin alone which is in line with sulfonylurea’s poor CV safety profile. As stated, for DPP-4 inhibitors and pioglitazone there are data showing both good and bad safety profile, so one could expect association with COVID-19 death go both ways, therefore our results are not surprising. Adding SGLT-2 inhibitors in our study was COVID-19 death-neutral which is also in line with SGLT-2 inhibitors’ CV safety profile.

Surprise in our study comes from combination of metformin+GLP-1 agonists. Adding GLP-1 agonist to metformin lead to several times increased risk of COVID-19 deaths in comparison to metformin alone. As stated above, GLP-1 agonists are generally considered to have a good CV safety profile. However, more detailed research does reveal some interesting facts and doubts:

First, clinical guidelines do seem to be more cautious in stating GLP-1 agonists are all CV beneficial as a class than for example when it comes to SGLT-2 inhibitors.

It should be noted that current clinical guidelines for diabetes type 2 treatment do state that duraglutide, liraglutide, and semaglutide are beneficial when it comes to MACE prevention, but also state exenatide and lixisenatide are MACE neutral. Also, they state the whole class is neutral when it comes to heart failure. SGLT-2 inhibitors they do regard beneficial for both indications, MACE and HF prevention in the guidelines [34]. One could argue that there are some residual caution in the guidelines towards stating that GLP-1 agonists are completely CV beneficial.

Second, European Medicines Agency refused to approve indication of reducing the risk of major heart and circulation problems in adults with a history of serious cardiovascular disease (such as a heart attack, stroke or poor blood flow to the limbs) and a BMI of 27 kg/m 2 or greater for GLP-1 agonists.

While the FDA has approved GLP-1 agonists indication of reducing the risk of major heart and circulation problems in adults with a history of serious cardiovascular disease (such as a heart attack, stroke or poor blood flow to the limbs) and a BMI of 27 kg/m^2^ or greater in overweight, EMA has rejected it stating that the use of semaglutide in this group of people is already covered by the approved indication for weight management [56].

Third, there are published data of GLP-1 agonists being associated with an increase in heart rate and other arrhythmia.

Despite the reported cardiovascular benefits, the prescription of GLP-1 agonists by cardiologists has so far remained low. There are several possible reasons for this – clinical inertia, a sense that prescribing these drugs require endocrinologist expertise, or the subcutaneous mode of administration. However, another possibility may be uncertainties related to GLP-1 agonists administration increasing heart rate and the latter having been associated with cardiovascular mortality [57]. A resting heart rate increase of 5 bpm has been associated with a 17% increase in cardiovascular mortality, with estimates exceeding 20% for heart rate increases of 10 bpm. GLP-1 agonist CV outcome trials have documented that GLP-1 agonists increase heart rate by 0.1–5.7 bpm, with an average increase of 2.4 bpm but also during continuous monitoring increases as high as 6−10 bpm. Increased heart rate might be a problem in patients with reduced ejection fraction. A study on large animal model showed that GLP-1 has direct chronotropic effects on the heart mediated by GLP-1 receptors in pacemaker cells of the sinus node [57].

Ussher et Drucker in their review paper conclude that there are studies that show that GLP-1 agonists increase heart rate and that this might not be beneficial in individuals with severe left ventricular dysfunction, reduced ejection fraction and/or a history of repeated hospitalization for heart failure [58].

A meta-analysis of randomized controlled trials from 2022 showed that there was increased risk of atrial fibrillation with dulaglutide (RR 1.40) while an inverse trend with oral semaglutide (RR 0.43) was seen. Higher doses of GLP-1 agonists (RR 1.63) and higher baseline BMI (RR 1.60) might have significantly increased the risk of ventricular arrhythmias [59].

Fourth, FAD and MHRA raised concerns over certain safety signals for GLP-1 agonists.

Data from spontaneous adverse events reporting schemes from the USA and the UK show some potentially concerning safety signals for GLP-1 agonists. Data from the Medicines and Healthcare Products Regulatory Agency (MHRA) show that up to 31 January 2025 there were 22 deaths from adverse reactions associated with use of GLP-1 agonists for weight loss and 60 linked to use for treating type 2 diabetes [60]. Bhattacharyya M et al conducted an analysis of the FDA Adverse Events Reporting database (FAERS) and calculated the reporting odds ratio (ROR) for mortality and serious adverse events for each GLP-1 agonist compared to the combined group of all other GLP-1 agonists. The ROR represents the ratio of the odds of reporting a complication for a particular medication relative to the odds of reporting the same complication for the remaining medications in the same therapeutic class. An ROR greater than 1.0 indicates higher odds of the complication being reported for the drug of interest compared to the class comparator. They found that disproportionality analyses revealed statistically significant elevated signals for mortality with the earliest approved GLP-1 agonists: exenatide (ROR = 2.20) and liraglutide [61]. Although pharmacovigilance data cannot establish causation, they are important tools for postmarketing surveillance.

In our database, patients who take metformin+GLP-1 agonists have higher proportions of CV disease than patients taking other drugs with metformin which, considering the previously elaborated points, could at least partly explain the poor results in our patients. In comparison to patients taking metformin alone or metformin+other drug, more patients taking metformin+GLP-1 agonist have at least one CV disease (metformin+pioglitazone have lowest percentage of 81.30%, metformin+GLP-1 agonist 88.7%). This is especially true for arterial hypertension (lowest metformin+DPP-4 inhibitor 77.20%, metformin+GLP-1 agonist 85.10%).

Our analysis showed that patients taking metformin+GLP-1 agonist have longer diabetes duration in comparison to metformin alone and other combinations except sulfonylurea combination (metformin alone 25.40%, metformin+GLP-1 agonist 51.10%), but tend to be younger than other groups (the oldest are metformin+sulfonylurea group (mean age ± SD) 70.55 ± 10.32, while metformin+GLP-1 agonist are 60.05 ± 10.10). Longer diabetes duration plus younger age could mean that their diabetes mellitus is more severe so this also might partly explain poor results for patients taking metformin+GLP-1 agonist.

It has to be noted that all these risks are very large – patients aged ≥76 have up to 7.51 times greater risk of COVID-19 death if they are taking metformin+GLP-1 agonist in comparison to metformin alone.

When we take presented data together with the presented information about potential increases in heart rate, arrhythmia and pharmacovigilance data, maybe we could say that it remains to be seen what will be happening with GLP-1 agonists in context of CV safety, especially in context of acute infectious diseases like COVID-19 which present strain on CV system.

It is to be noted that our patients taking metformin+GLP-1 agonist have more lower respiratory tract chronic and obstructive diseases than patients taking other combinations or metformin alone (metformin+SGLT-2 inhibitor group have lowest percentage of 13.40%, while metformin+GLP-1 agonist 21.10%). This could have also contributed to the poor result of metformin+GLP-1 agonist combination.

Finally, in all our models R^2^ (coefficient of determination) did not exceed 30% which means we have explained up to 30% of COVID-19 deaths with variables we used. The R^2^ would be higher had we been able to use HbA1c% and BMI data. However, this is not to say we used small number of variables, but it is to say there are still many factors affecting COVID-19 death we do not know about.

We can say that adding GLP-1 agonists to metformin could require caution in certain patient groups. Furter research is needed – probably each member of GLP-1 agonist class should be researched separately since it seems that they do not all share same class effects when it comes to CV safety.

### Study limitations

There are several limitations to this study.

One is its retrospective and observational design.

As with all observational studies, confounding by indication cannot be entirely excluded. Treatment selection, monotherapy vs combination, reflects complex clinical decision-making based on diabetes severity, metabolic control, comorbidity patterns – factors not all fully captured in registry data. However, several features of our study design mitigate this concern. First, we adjusted for 18 clinically relevant covariates including indicators of diabetes mellitus severity (diabetes duration, multiple cardiovascular comorbidities, chronic kidney disease, ACEI/ARB use), SARS-CoV-2 vaccination status, age and sex. Second, we employed IPTW with stabilized weights, which creates a pseudo-population where treatment assignment is independent of measured confounders. The achievement of excellent covariate balance (SMD < 0.1) for 4 out of 5 combinations, i.e., all besides metformin+GLP-1 agonist, after weighting suggests measured confounding was adequately addressed. Third, while HbA1c and BMI were unavailable, diabetes duration, multiple cardiovascular comorbidities, chronic kidney disease, ACEI/ARB use served as a proxy for disease severity. Nevertheless, unmeasured confounding remains a possibility and our findings should be interpreted as associations rather than definitive causal relationships.

Comparing combination therapies to monotherapy can also be biased since patients using combination therapy have more advanced diabetes. This was done reflecting the clinical decision point when escalation beyond first-line therapy becomes necessary. So we believe this can help clinicians in making decisions which antidiabetic to add to metformin once monotherapy is not enough. Nevertheless, comparing combination therapies directly to each other in future studies could provide complementary insights into relative safety profiles.

Other limitations are that part of the population was excluded from the analysis due to lack of reliable medication data.

Also, HbA1c and BMI could not be included in logistic regression models due to insufficient data. Not having access to patients’ medical records and missing clinical data such as BMI and HbA1c is a known drawback of working with public health databases [62]. Some of the other published studies of association of antidiabetics and COVID-19 outcomes performed on large national datasets also lack these variables such as study done on Swedish registry by Ferrannini et al [63]. In our study, inclusion of proxy for disease severity and cumulative glycemic exposure (diabetes duration, multiple cardiovascular comorbidities, chronic kidney disease, ACEI/ARB use) might partially mitigate absence of HbA1c and BMI. Nevertheless, the lack of these direct measures limits our ability to fully disentangle metabolic control from medication effects, and this limitation is particularly relevant for the metformin+GLP-1 agonist findings, where BMI is often a key factor in prescribing decisions. As shown by the R^2^, our regression models did explain up to 30% of variance in COVID-19 deaths. R² would have been somewhat higher with HbA1c and BMI data, though the consistent direction and magnitude of effects across weighted and unweighted analyses suggest our main findings are reasonably robust.

Further on, our drug exposure definition was binary (yes/no) and did not account for dose, persistence, or treatment changes over time. However, our drug exposure definition was more sophisticated than just yes/no categorization since anti-diabetic drug intake was defined as a prescription filled at least twice within eight months before the SARS-CoV-2 infection or COVID-19 outcome (or before a primary care visit with diabetes diagnosis for those without outcomes). This ensured that all patients who were defined as antidiabetics-users had to take the drug for some time and that patients who just tried the drug and stopped its use were not regarded as antidiabetics users. Nevertheless, future studies with detailed prescription records including specific drugs, dose titration, and treatment persistence would strengthen causal inference.

Our regression models were adjusted for many confounders and also, we additionally mitigated effects of confounders by IPTW. However, for metformin+GLP-1 agonist combination, 2 of 18 variables were imbalanced, age and arterial hypertension, even after weighing and this is a limitation. The metformin+GLP-1 agonist combination results should therefore be interpreted with special caution and should be considered hypothesis-generating rather than definitive. The evidence we present from multiple independent sources (mechanistic concerns about heart rate effects, arrhythmia signals, FDA/MHRA pharmacovigilance data, EMA regulatory decisions) supports the need for further investigation but does not overcome the methodological limitation in our own analysis.

Another limitation is small event numbers. For some drug combinations, particularly metformin+GLP-1 agonist (n = 22 deaths) and metformin+pioglitazone (n = 15 deaths), the absolute number of COVID-19 death events was modest. While adequate sample sizes existed in the denominators (2,499 and 2,629 patients respectively), small event numbers increase statistical uncertainty and widen confidence intervals, as reflected in our reported 95% CIs. However, our analytical approach demonstrated ability to detect null findings even with small event numbers (metformin+pioglitazone n = 15 events and metformin+SGLT-2 inhibitor n = 33 events showed no increased risk), indicating we are not systematically biased toward positive findings with small samples. Nevertheless, the modest event count for metformin+GLP-1 agonist contributes to the need for cautious interpretation and replication in larger cohorts.

These findings should be interpreted as hypothesis-generating signals from real-world data rather than evidence of causal drug effects.

## Supporting information

S1 Table Group differences for metformin+DPP-4 inhibitors vs metformin alone before and after weighing. (DOCX)

S2 Table Group differences for metformin+sulfonylurea vs metformin alone before and after weighing. (DOCX)

S3 Table Group differences for metformin+SGLT-2 inhibitor vs metformin alone before and after weighing. (DOCX)

S4 Table Group differences for metformin+pioglitazone vs metformin alone before and after weighing. (DOCX)

S5 Table Group differences for metformin+GLP-1 agonist vs metformin alone before and after weighing. (DOCX)

S6 Table Logistic regression for metformin+DPP-4 inhibitor vs metformin only prior weighing. (DOCX)

S7 Table Logistic regression for metformin+DPP-4 inhibitor vs metformin only after weighing. (DOCX)

S8 Table Logistic regression for metformin+sulfonylurea vs metformin only prior weighing. (DOCX)

S9 Table Logistic regression for metformin+sulfonylurea vs metformin only after weighing. (DOCX)

S10 Table Logistic regression for metformin+SGLT-2 inhibitor vs metformin only prior weighing. (DOCX)

S11 Table Logistic regression for metformin+SGLT-2 inhibitor vs metformin only after weighing. (DOCX)

S12 Table Logistic regression for metformin+pioglitazone vs metformin only prior weighing. (DOCX)

S13 Table Logistic regression for metformin+pioglitazone vs metformin only after weighing. (DOCX)

S14 Table Logistic regression for metformin+GLP-1 agonist vs metformin only prior weighing. (DOCX)

S15 Table Logistic regression for metformin+GLP-1 agonist vs metformin only after weighing. (DOCX)
