# Supplementary material for: Identifying high-risk combinations of metformin during COVID-19
Source: PLoS One. 2026 Mar 4;21(3):e0343979. doi: 10.1371/journal.pone.0343979 (PMC12959685; doi:10.1371/journal.pone.0343979)
Supplement: S1 Table — (DOCX) [file pone.0343979.s001.docx]

S1 Table Group differences for metformin+DPP-4 inhibitors vs metformin alone before and after weighing

| prior weighing | |  |  |  |  | after weighing | |  |  |  |
| --- | --- | --- | --- | --- | --- | --- | --- | --- | --- | --- |
|  | combination | | metformin alone | | SMD | combination | | metformin alone | | SMD |
| N | 26488 |  | 85553 |  |  | 26501 |  | 85546 |  |  |
| variable |  |  |  |  |  |  |  |  |  |  |
| age, mean±SD | 65.06±10.45 | | 65.84±11.80 | | 0.068 | 65.61±10.42 | | 65.66±11.86 | | 0.004 |
|  | N |  | N |  |  | N |  | N |  |  |
| diabetes duration>7 years | 11563 | 43.70% | 21758 | 25.40% | 0.062 | 7835 | 29.60% | 25416 | 29.70% | 0.002 |
| sex, female | 12003 | 45.30% | 44051 | 51.50% | 0.399 | 13224 | 49.90% | 42780 | 50.00% | -0.009 |
| ACEI | 13553 | 51.20% | 43356 | 50.70% | 0.01 | 13413 | 50.60% | 43439 | 50.80% | -0.004 |
| ARB | 824 | 3.10% | 2754 | 3.20% | 0.006 | 830 | 3.10% | 2725 | 3.20% | 0.002 |
| SARS-CoV-2 vaccination | 20056 | 75.70% | 64314 | 75.20% | -0.01 | 19825 | 74.80% | 64385 | 75.30% | -0.011 |
| SARS-CoV-2 positivity | 3873 | 14.60% | 11752 | 13.70% | -0.027 | 3770 | 14.20% | 11829 | 13.80% | 0.012 |
| COVID-19 hospitalization | 977 | 3.70% | 2885 | 3.40% | -0.015 | 980 | 3.70% | 2898 | 3.40% | 0.016 |
| COVID-19 death | 233 | 0.90% | 692 | 0.80% | -0.011 | 237 | 0.90% | 696 | 0.80% | 0.007 |
| cancer | 2300 | 8.70% | 7648 | 8.90% | -0.007 | 2362 | 8.90% | 7598 | 8.90% | 0 |
| arterial hypertension | 20456 | 77.20% | 66717 | 78.00% | -0.019 | 20552 | 77.60% | 66540 | 77.80% | -0.005 |
| ischemic heart disease | 3342 | 12.60% | 10327 | 12.10% | -0.015 | 3180 | 12.00% | 10421 | 12.20% | -0.005 |
| cardiomyopathy | 1124 | 4.20% | 3918 | 4.60% | -0.02 | 1199 | 4.50% | 3851 | 4.50% | 0 |
| cerebrovascular diseases | 1528 | 5.80% | 4652 | 5.40% | -0.017 | 1461 | 5.50% | 4719 | 5.50% | 0 |
| circulatory diseases other than hypertension | 8981 | 33.90% | 29829 | 34.90% | -0.021 | 9140 | 34.50% | 29617 | 34.60% | -0.002 |
| lower respiratory tract chronic diseases | 2416 | 9.10% | 8488 | 9.90% | -0.027 | 2572 | 9.70% | 8322 | 9.70% | 0 |
| other obstructive lung diseases | 1258 | 4.70% | 4335 | 5.10% | -0.018 | 1328 | 5.00% | 4273 | 5.00% | 0 |
| chronic kidney disease | 399 | 1.50% | 1062 | 1.20% |  | 338 | 1.30% | 1111 | 1.30% | 0 |

SD=standard deviation; DPP-4 = Dipeptidyl peptidase 4, SGLT-2 = Sodium-glucose co-transporter 2, GLP-1 = Glucagon-like peptide-1, ACEI= Angiotensin-converting enzyme inhibitors, ARB=Angiotensin receptor blockers, COVID-19= coronavirus disease 19, SARS-CoV-2= Severe acute respiratory syndrome coronavirus 2
