# Supplementary material for: Identifying high-risk combinations of metformin during COVID-19
Source: PLoS One. 2026 Mar 4;21(3):e0343979. doi: 10.1371/journal.pone.0343979 (PMC12959685; doi:10.1371/journal.pone.0343979)
Supplement: S2 Table — (DOCX) [file pone.0343979.s002.docx]

S2 Table Group differences for metformin+sulfonylurea vs metformin alone before and after weighing

| prior weighing | |  |  |  |  | after weighing | |  |  |  |
| --- | --- | --- | --- | --- | --- | --- | --- | --- | --- | --- |
|  | combination | | metformin alone | | SMD | combination | | metformin alone | | SMD |
| N | 17722 |  | 85553 |  |  | 17599 |  | 85588 |  |  |
| variable |  |  |  |  |  |  |  |  |  |  |
| age, mean±SD | 70.55±10.32 | | 65.84±11.80 | | -0.025 | 66.96±11.10 | | 66.67±11.77 | | -0.025 |
|  | N |  | N |  |  | N |  | N |  |  |
| diabetes duration>7 years | 11480 | 64.80% | 21758 | 25.40% | -0.006 | 5713 | 32.50% | 27573 | 32.20% | -0.006 |
| sex, female | 8725 | 49.20% | 44051 | 51.50% | -0.014 | 8876 | 50.40% | 43720 | 51.10% | -0.014 |
| ACEI | 9777 | 55.20% | 43356 | 50.70% | -0.006 | 9100 | 51.70% | 44035 | 51.40% | -0.006 |
| ARB | 624 | 3.50% | 2754 | 3.20% | 0.006 | 566 | 3.20% | 2796 | 3.30% | 0.006 |
| SARS-CoV-2 vaccination | 12231 | 69.00% | 64314 | 75.20% | 0.009 | 12947 | 73.60% | 63377 | 74.00% | 0.009 |
| SARS-CoV-2 positivity | 2488 | 14.00% | 11752 | 13.70% | 0.009 | 2383 | 13.50% | 11804 | 13.80% | 0.009 |
| COVID-19 hospitalization | 985 | 5.60% | 2885 | 3.40% | -0.046 | 791 | 4.50% | 3076 | 3.60% | -0.046 |
| COVID-19 death | 273 | 1.50% | 692 | 0.80% | -0.02 | 190 | 1.10% | 782 | 0.90% | -0.02 |
| cancer | 1708 | 9.60% | 7648 | 8.90% | -0.007 | 1644 | 9.30% | 7768 | 9.10% | -0.007 |
| arterial hypertension | 14586 | 82.30% | 66717 | 78.00% | -0.01 | 13926 | 79.10% | 67383 | 78.70% | -0.01 |
| ischemic heart disease | 2504 | 14.10% | 10327 | 12.10% | 0 | 2182 | 12.40% | 10629 | 12.40% | 0 |
| cardiomyopathy | 1155 | 6.50% | 3918 | 4.60% | -0.014 | 919 | 5.20% | 4228 | 4.90% | -0.014 |
| cerebrovascular diseases | 1270 | 7.20% | 4652 | 5.40% | -0.004 | 1042 | 5.90% | 4922 | 5.80% | -0.004 |
| circulatory diseases other than hypertension | 6873 | 38.80% | 29829 | 34.90% | -0.006 | 6315 | 35.90% | 30437 | 35.60% | -0.006 |
| lower respiratory tract chronic diseases | 1719 | 9.70% | 8488 | 9.90% | -0.007 | 1772 | 10.10% | 8467 | 9.90% | -0.007 |
| other obstructive lung diseases | 965 | 5.40% | 4335 | 5.10% | -0.009 | 941 | 5.30% | 4401 | 5.10% | -0.009 |
| chronic kidney disease | 333 | 1.90% | 1062 | 1.20% | -0.009 | 238 | 1.40% | 1155 | 1.30% | -0.009 |

SD=standard deviation; DPP-4 = Dipeptidyl peptidase 4, SGLT-2 = Sodium-glucose co-transporter 2, GLP-1 = Glucagon-like peptide-1, ACEI= Angiotensin-converting enzyme inhibitors, ARB=Angiotensin receptor blockers, COVID-19= coronavirus disease 19, SARS-CoV-2= Severe acute respiratory syndrome coronavirus 2
