# Supplementary material for: Identifying high-risk combinations of metformin during COVID-19
Source: PLoS One. 2026 Mar 4;21(3):e0343979. doi: 10.1371/journal.pone.0343979 (PMC12959685; doi:10.1371/journal.pone.0343979)
Supplement: S3 Table — (DOCX) [file pone.0343979.s003.docx]

S3 Table Group differences for metformin+SGLT-2 inhibitor vs metformin alone before and after weighing

| prior weighing | |  |  |  |  | after weighing | |  |  |  |
| --- | --- | --- | --- | --- | --- | --- | --- | --- | --- | --- |
|  | combination | | metformin alone | | SMD | combination | | metformin alone | | SMD |
| N | 6123 |  | 85553 |  |  | 6075 |  | 85555 |  |  |
| variable |  |  |  |  |  |  |  |  |  |  |
| age, mean±SD | 60.46±10.28 | | 65.84±11.80 | | 0.4595 |  |  |  |  | 0.057 |
|  | N |  | N |  |  | N |  | N |  |  |
| diabetes duration>7 years | 2141 | 35.00% | 21758 | 25.40% | 0.2102 | 1423 | 23.40% | 22287 | 26.00% | 0.03 |
| sex, female | 1926 | 31.50% | 44051 | 51.50% | 0.4145 | 3140 | 51.70% | 42920 | 50.20% | 0.031 |
| ACEI | 3223 | 52.60% | 43356 | 50.70% | 0.038 | 2996 | 49.30% | 43458 | 50.80% | 0 |
| ARB | 211 | 3.40% | 2754 | 3.20% | 0.0112 | 193 | 3.20% | 2768 | 3.20% | 0.044 |
| SARS-CoV-2 vaccination | 4921 | 80.40% | 64314 | 75.20% | 0.1254 | 4606 | 75.80% | 64616 | 75.50% | 0.102 |
| SARS-CoV-2 positivity | 983 | 16.10% | 11752 | 13.70% | 0.0674 | 928 | 15.30% | 11801 | 13.80% | 0.002 |
| COVID-19 hospitalization | 181 | 3.00% | 2885 | 3.40% | 0.0227 | 192 | 3.20% | 2856 | 3.30% | 0.021 |
| COVID-19 death | 33 | 0.50% | 692 | 0.80% | 0.0373 | 39 | 0.60% | 676 | 0.80% | 0.007 |
| cancer | 394 | 6.40% | 7648 | 8.90% | 0.0942 | 523 | 8.60% | 7504 | 8.80% | 0.045 |
| arterial hypertension | 4670 | 76.30% | 66717 | 78.00% | 0.0405 | 4604 | 75.80% | 66604 | 77.80% | 0.012 |
| ischemic heart disease | 1341 | 21.90% | 10327 | 12.10% | 0.2631 | 750 | 12.30% | 10878 | 12.70% | 0.023 |
| cardiomyopathy | 382 | 6.20% | 3918 | 4.60% | 0.0708 | 247 | 4.10% | 4006 | 4.70% | 0.017 |
| cerebrovascular diseases | 311 | 5.10% | 4652 | 5.40% | 0.0135 | 291 | 4.80% | 4628 | 5.40% | 0.031 |
| circulatory diseases other than hypertension | 2464 | 40.20% | 29829 | 34.90% | 0.1096 | 2052 | 33.80% | 30121 | 35.20% | 0.003 |
| lower respiratory tract chronic diseases | 549 | 9.00% | 8488 | 9.90% | 0.0308 | 610 | 10.00% | 8436 | 9.90% | 0.009 |
| other obstructive lung diseases | 271 | 4.40% | 4335 | 5.10% | 0.0329 | 323 | 5.30% | 4299 | 5.00% | 0.005 |
| chronic kidney disease | 70 | 1.10% | 1062 | 1.20% | 0.0094 | 76 | 1.30% | 1056 | 1.20% | 0.007 |

SD=standard deviation; DPP-4 = Dipeptidyl peptidase 4, SGLT-2 = Sodium-glucose co-transporter 2, GLP-1 = Glucagon-like peptide-1, ACEI= Angiotensin-converting enzyme inhibitors, ARB=Angiotensin receptor blockers, COVID-19= coronavirus disease 19, SARS-CoV-2= Severe acute respiratory syndrome coronavirus 2
