# Supplementary material for: Identifying high-risk combinations of metformin during COVID-19
Source: PLoS One. 2026 Mar 4;21(3):e0343979. doi: 10.1371/journal.pone.0343979 (PMC12959685; doi:10.1371/journal.pone.0343979)
Supplement: S4 Table — (DOCX) [file pone.0343979.s004.docx]

S4 Table Group differences for metformin+pioglitazone vs metformin alone before and after weighing

| prior weighing | |  |  |  |  | after weighing | |  |  |  |
| --- | --- | --- | --- | --- | --- | --- | --- | --- | --- | --- |
|  | combination | | metformin alone | | SMD | combination | | metformin alone | | SMD |
| N | 2629 |  | 85553 |  |  | 2625 |  | 85553 |  |  |
| variable |  |  |  |  |  |  |  |  |  |  |
| age, mean±SD | 62.82±10.62 | | 65.84±11.80 | | 0.257 | 65.10±10.40 | | 65.75±11.84 | | 0.058 |
|  | N |  | N |  |  | N |  | N |  |  |
| diabetes duration>7 years | 1073 | 40.80% | 21758 | 25.40% | 0.331 | 649 | 24.70% | 22149 | 25.90% | 0.028 |
| sex, female | 1281 | 48.70% | 44051 | 51.50% | 0.055 | 1347 | 51.30% | 43981 | 51.40% | 0.002 |
| ACEI | 1410 | 53.60% | 43356 | 50.70% | 0.059 | 1316 | 50.10% | 43430 | 50.80% | 0.014 |
| ARB | 82 | 3.10% | 2754 | 3.20% | 0.006 | 80 | 3.00% | 2751 | 3.20% | 0.012 |
| SARS-CoV-2 vaccination | 2021 | 76.90% | 64314 | 75.20% | 0.04 | 1976 | 75.30% | 64357 | 75.20% | 0.002 |
| SARS-CoV-2 positivity | 370 | 14.10% | 11752 | 13.70% | 0.01 | 342 | 13.00% | 11772 | 13.80% | 0.024 |
| COVID-19 hospitalization | 87 | 3.30% | 2885 | 3.40% | 0.004 | 97 | 3.70% | 2877 | 3.40% | 0.016 |
| COVID-19 death | 15 | 0.60% | 692 | 0.80% | 0.029 | 15 | 0.60% | 688 | 0.80% | 0.024 |
| cancer | 170 | 6.50% | 7648 | 8.90% | 0.093 | 216 | 8.20% | 7584 | 8.90% | 0.025 |
| arterial hypertension | 2047 | 77.90% | 66717 | 78.00% | 0.003 | 2012 | 76.60% | 66712 | 78.00% | 0.034 |
| ischemic heart disease | 197 | 7.50% | 10327 | 12.10% | 0.155 | 321 | 12.20% | 10210 | 11.90% | 0.009 |
| cardiomyopathy | 76 | 2.90% | 3918 | 4.60% | 0.089 | 126 | 4.80% | 3875 | 4.50% | 0.014 |
| cerebrovascular diseases | 147 | 5.60% | 4652 | 5.40% | 0.007 | 135 | 5.10% | 4656 | 5.40% | 0.014 |
| circulatory diseases other than hypertension | 721 | 27.40% | 29829 | 34.90% | 0.161 | 913 | 34.80% | 29639 | 34.60% | 0.004 |
| lower respiratory tract chronic diseases | 239 | 9.10% | 8488 | 9.90% | 0.028 | 251 | 9.60% | 8467 | 9.90% | 0.01 |
| other obstructive lung diseases | 125 | 4.80% | 4335 | 5.10% | 0.014 | 126 | 4.80% | 4327 | 5.10% | 0.014 |
| chronic kidney disease | 30 | 1.10% | 1062 | 1.20% | 0.009 | 31 | 1.20% | 1059 | 1.20% | 0 |

SD=standard deviation; DPP-4 = Dipeptidyl peptidase 4, SGLT-2 = Sodium-glucose co-transporter 2, GLP-1 = Glucagon-like peptide-1, ACEI= Angiotensin-converting enzyme inhibitors, ARB=Angiotensin receptor blockers, COVID-19= coronavirus disease 19, SARS-CoV-2= Severe acute respiratory syndrome coronavirus 2
