# Supplementary material for: Identifying high-risk combinations of metformin during COVID-19
Source: PLoS One. 2026 Mar 4;21(3):e0343979. doi: 10.1371/journal.pone.0343979 (PMC12959685; doi:10.1371/journal.pone.0343979)
Supplement: S5 Table — (DOCX) [file pone.0343979.s005.docx]

S5 Table Group differences for metformin+GLP-1 agonist vs metformin alone before and after weighing

| prior weighing |  |  |  |  |  | after weighing with sw | | |  |  |
| --- | --- | --- | --- | --- | --- | --- | --- | --- | --- | --- |
|  | combination | | metformin alone | | SMD | combination | | metformin alone | | SMD |
| N | 2499 |  | 85553 |  |  | 2412 |  | 85597 |  |  |
| variable |  |  |  |  |  |  |  |  |  |  |
| age, mean±SD | 60.05±10.01 | | 65.84±11.80 | | 0.492 | 63.05±9.39 | | 65.67±11.90 | | **0.221** |
|  | N |  | N |  |  | N |  | N |  |  |
| diabetes duration>7 years | 1277 | 51.10% | 21758 | 25.40% | -0.584 | 551 | 22.80% | 22393 | 26.20% | 0.079 |
| sex, female | 1405 | 56.20% | 44051 | 51.50% | 0.095 | 1203 | 49.90% | 44193 | 51.60% | -0.034 |
| ACEI | 1263 | 50.50% | 43356 | 50.70% | -0.003 | 1169 | 48.50% | 43371 | 50.70% | -0.044 |
| ARB | 112 | 4.50% | 2754 | 3.20% | 0.071 | 66 | 2.70% | 2784 | 3.30% | -0.035 |
| SARS-CoV-2 vaccination | 2030 | 81.20% | 64314 | 75.20% | 0.141 | 1822 | 75.60% | 64498 | 75.30% | 0.007 |
| SARS-CoV-2 positivity | 391 | 15.60% | 11752 | 13.70% | 0.055 | 403 | 16.70% | 11782 | 13.80% | 0.081 |
| COVID-19 hospitalization | 83 | 3.30% | 2885 | 3.40% | -0.003 | 91 | 3.80% | 2869 | 3.40% | 0.022 |
| COVID-19 death | 22 | 0.90% | 692 | 0.80% | 0.008 | 38 | 1.60% | 684 | 0.80% | 0.074 |
| cancer | 172 | 6.90% | 7648 | 8.90% | -0.072 | 199 | 8.30% | 7601 | 8.90% | -0.021 |
| arterial hypertension | 2126 | 85.10% | 66717 | 78.00% | 0.172 | 1684 | 69.80% | 66915 | 78.20% | **-0.192** |
| ischemic heart disease | 350 | 14.00% | 10327 | 12.10% | 0.059 | 269 | 11.20% | 10376 | 12.10% | -0.028 |
| cardiomyopathy | 134 | 5.40% | 3918 | 4.60% | 0.037 | 93 | 3.90% | 3939 | 4.60% | -0.035 |
| cerebrovascular diseases | 151 | 6.00% | 4652 | 5.40% | 0.027 | 109 | 4.50% | 4666 | 5.50% | -0.046 |
| circulatory diseases other than hypertension | 970 | 38.80% | 29829 | 34.90% | 0.083 | 781 | 32.40% | 29933 | 35.00% | -0.055 |
| lower respiratory tract chronic diseases | 353 | 14.10% | 8488 | 9.90% | 0.14 | 233 | 9.70% | 8594 | 10.00% | -0.01 |
| other obstructive lung diseases | 174 | 7.00% | 4335 | 5.10% | 0.086 | 112 | 4.60% | 4382 | 5.10% | -0.023 |
| chronic kidney disease | 54 | 2.20% | 1062 | 1.20% | 0.082 | 28 | 1.20% | 1085 | 1.30% | -0.009 |

SD=standard deviation; DPP-4 = Dipeptidyl peptidase 4, SGLT-2 = Sodium-glucose co-transporter 2, GLP-1 = Glucagon-like peptide-1, ACEI= Angiotensin-converting enzyme inhibitors, ARB=Angiotensin receptor blockers, COVID-19= coronavirus disease 19, SARS-CoV-2= Severe acute respiratory syndrome coronavirus 2
