# Supplementary material for: Identifying high-risk combinations of metformin during COVID-19
Source: PLoS One. 2026 Mar 4;21(3):e0343979. doi: 10.1371/journal.pone.0343979 (PMC12959685; doi:10.1371/journal.pone.0343979)
Supplement: S6 Table — (DOCX) [file pone.0343979.s006.docx]

S6 Table Logistic regression for metformin+DPP-4 inhibitor vs metformin only prior weighing

Nagelkerke R Square 0.229

Sig.<0.001

|  | B | S.E. | Wald | df | Sig. | Exp(B) | 95% C.I.for EXP(B) | |
| --- | --- | --- | --- | --- | --- | --- | --- | --- |
|  |  |  |  |  |  |  | Lower | Upper |
| Age | 0.067 | 0.004 | 361.215 | 1 | <,001 | 1.069 | 1.062 | 1.077 |
| Diabetes duration shorter than 7 years(1) | -0.267 | 0.101 | 6.972 | 1 | 0.008 | 0.765 | 0.628 | 0.933 |
| Sex (female) | -0.825 | 0.071 | 135.029 | 1 | <,001 | 0.438 | 0.381 | 0.504 |
| ACEI | -0.045 | 0.072 | 0.385 | 1 | 0.535 | 0.956 | 0.831 | 1.101 |
| ARB | -0.231 | 0.205 | 1.269 | 1 | 0.26 | 0.794 | 0.532 | 1.186 |
| Vaccination p1 | -0.918 | 0.148 | 38.719 | 1 | <,001 | 0.399 | 0.299 | 0.533 |
| Vaccination p2 | -1.69 | 0.18 | 88.619 | 1 | <,001 | 0.185 | 0.13 | 0.262 |
| Vaccination b1 | -2.465 | 0.393 | 39.34 | 1 | <,001 | 0.085 | 0.039 | 0.184 |
| Neoplasm | 0.145 | 0.104 | 1.962 | 1 | 0.161 | 1.156 | 0.944 | 1.416 |
| Arterial hypertension | 0.28 | 0.105 | 7.136 | 1 | 0.008 | 1.323 | 1.077 | 1.625 |
| Ishemic heart disease | -0.018 | 0.1 | 0.033 | 1 | 0.856 | 0.982 | 0.808 | 1.194 |
| Cardiomyopathy | 0.045 | 0.117 | 0.146 | 1 | 0.703 | 1.046 | 0.831 | 1.315 |
| Cerebrovscular diseases | -0.033 | 0.12 | 0.078 | 1 | 0.781 | 0.967 | 0.765 | 1.223 |
| Circulatory diseases except hypertension | 0.249 | 0.086 | 8.286 | 1 | 0.004 | 1.282 | 1.083 | 1.519 |
| Chronic lower respiratory diseases | 0.101 | 0.156 | 0.42 | 1 | 0.517 | 1.107 | 0.815 | 1.503 |
| Other chronic obstructive lung diseases | 0.336 | 0.185 | 3.286 | 1 | 0.07 | 1.399 | 0.973 | 2.01 |
| Chronic kidney disease | 0.36 | 0.182 | 3.923 | 1 | 0.048 | 1.434 | 1.004 | 2.048 |
| Metformin+DPP-4_vs_metformin only | 0.178 | 0.079 | 5.14 | 1 | 0.023 | 1.195 | 1.024 | 1.395 |
| Constant | -8.061 | 0.281 | 822.71 | 1 | <,001 | 0 |  |  |

DPP-4 = Dipeptidyl peptidase 4, ACEI= Angiotensin-converting enzyme inhibitors, ARB=Angiotensin receptor blockers
