# Supplementary material for: Identifying high-risk combinations of metformin during COVID-19
Source: PLoS One. 2026 Mar 4;21(3):e0343979. doi: 10.1371/journal.pone.0343979 (PMC12959685; doi:10.1371/journal.pone.0343979)
Supplement: S7 Table — (DOCX) [file pone.0343979.s007.docx]

S7 Table Logistic regression for metformin+DPP-4 inhibitor vs metformin only after weighing

|  | B | S.E. | Wald | df | Sig. | Exp(B) | 95% C.I.for EXP(B) | |
| --- | --- | --- | --- | --- | --- | --- | --- | --- |
|  |  |  |  |  |  |  | Lower | Upper |
| Age | 0.064 | 0.004 | 322.03 | 1 | <,001 | 1.066 | 1.059 | 1.074 |
| Diabetes duration shorter than 7 years | -0.219 | 0.07 | 9.928 | 1 | 0.002 | 0.803 | 0.701 | 0.92 |
| Sex (female) | -0.795 | 0.071 | 126.974 | 1 | <,001 | 0.452 | 0.393 | 0.519 |
| ACEI | -0.036 | 0.071 | 0.251 | 1 | 0.617 | 0.965 | 0.839 | 1.11 |
| ARB | -0.251 | 0.206 | 1.486 | 1 | 0.223 | 0.778 | 0.519 | 1.165 |
| Vaccination p1 | -0.9 | 0.146 | 38.111 | 1 | <,001 | 0.407 | 0.306 | 0.541 |
| Vaccination p2 | -1.724 | 0.179 | 93.133 | 1 | <,001 | 0.178 | 0.126 | 0.253 |
| Vaccination b1 | -2.471 | 0.399 | 38.376 | 1 | <,001 | 0.084 | 0.039 | 0.185 |
| Neoplasm | 0.165 | 0.103 | 2.567 | 1 | 0.109 | 1.179 | 0.964 | 1.442 |
| Arterial hypertension | 0.283 | 0.105 | 7.261 | 1 | 0.007 | 1.327 | 1.08 | 1.629 |
| Ishemic heart disease | 0.019 | 0.099 | 0.037 | 1 | 0.847 | 1.019 | 0.84 | 1.238 |
| Cardiomyopathy | 0.075 | 0.116 | 0.418 | 1 | 0.518 | 1.078 | 0.859 | 1.352 |
| Cerebrovscular diseases | -0.062 | 0.121 | 0.268 | 1 | 0.605 | 0.939 | 0.742 | 1.19 |
| Circulatory diseases except hypertension | 0.25 | 0.086 | 8.39 | 1 | 0.004 | 1.284 | 1.084 | 1.52 |
| Chronic lower respiratory diseases | 0.125 | 0.155 | 0.653 | 1 | 0.419 | 1.133 | 0.837 | 1.535 |
| Other chronic obstructive lung diseases | 0.327 | 0.184 | 3.17 | 1 | 0.075 | 1.387 | 0.968 | 1.987 |
| Chronic kidney disease | 0.31 | 0.186 | 2.778 | 1 | 0.096 | 1.364 | 0.947 | 1.965 |
| Metformin+DPP-4_vs_metformin only | 0.167 | 0.077 | 4.664 | 1 | 0.031 | 1.182 | 1.016 | 1.376 |
| Constant | -7.97 | 0.272 | 857.571 | 1 | <,001 | 0 |  |  |

DPP-4 = Dipeptidyl peptidase 4, ACEI= Angiotensin-converting enzyme inhibitors, ARB=Angiotensin receptor blockers
