# Supplementary material for: Identifying high-risk combinations of metformin during COVID-19
Source: PLoS One. 2026 Mar 4;21(3):e0343979. doi: 10.1371/journal.pone.0343979 (PMC12959685; doi:10.1371/journal.pone.0343979)
Supplement: S8 Table — (DOCX) [file pone.0343979.s015.docx]

S15 Table Logistic regression for metformin+GLP-1 agonist vs metformin only after weighing

|  | B | S.E. | Wald | df | Sig. | Exp(B) | 95% C.I.for EXP(B) | |
| --- | --- | --- | --- | --- | --- | --- | --- | --- |
|  |  |  |  |  |  |  | Lower | Upper |
| Age | 0.066 | 0.004 | 267.972 | 1 | <,001 | 1.069 | 1.06 | 1.077 |
| Diabetes duration shorter than 7 years | -0.175 | 0.08 | 4.702 | 1 | 0.03 | 0.84 | 0.717 | 0.983 |
| Sex (female) | -0.878 | 0.08 | 120.234 | 1 | <,001 | 0.416 | 0.355 | 0.486 |
| ACEI | -0.098 | 0.081 | 1.463 | 1 | 0.227 | 0.907 | 0.774 | 1.063 |
| ARB | -0.172 | 0.223 | 0.593 | 1 | 0.441 | 0.842 | 0.543 | 1.304 |
| Vaccination p1 | -0.992 | 0.173 | 32.952 | 1 | <,001 | 0.371 | 0.264 | 0.52 |
| Vaccination p2 | -1.623 | 0.208 | 60.955 | 1 | <,001 | 0.197 | 0.131 | 0.297 |
| Vaccination b1 | -2.365 | 0.426 | 30.815 | 1 | <,001 | 0.094 | 0.041 | 0.217 |
| Neoplasm | 0.166 | 0.116 | 2.036 | 1 | 0.154 | 1.18 | 0.94 | 1.483 |
| Arterial hypertension | 0.357 | 0.12 | 8.789 | 1 | 0.003 | 1.429 | 1.129 | 1.809 |
| Ishemic heart disease | -0.145 | 0.115 | 1.6 | 1 | 0.206 | 0.865 | 0.691 | 1.083 |
| Cardiomyopathy | -0.044 | 0.134 | 0.11 | 1 | 0.74 | 0.957 | 0.736 | 1.243 |
| Cerebrovscular diseases | -0.078 | 0.138 | 0.319 | 1 | 0.572 | 0.925 | 0.705 | 1.213 |
| Circulatory diseases except hypertension | 0.311 | 0.096 | 10.505 | 1 | 0.001 | 1.365 | 1.131 | 1.649 |
| Chronic lower respiratory diseases | 0.246 | 0.169 | 2.13 | 1 | 0.144 | 1.279 | 0.919 | 1.78 |
| Other chronic obstructive lung diseases | 0.291 | 0.199 | 2.136 | 1 | 0.144 | 1.338 | 0.905 | 1.979 |
| Chronic kidney disease | 0.578 | 0.199 | 8.456 | 1 | 0.004 | 1.783 | 1.208 | 2.633 |
| Metformin+GLP-1_vs_metformin only | 1.096 | 0.177 | 38.514 | 1 | <,001 | 2.992 | 2.117 | 4.229 |
| Constant | -8.191 | 0.315 | 674.236 | 1 | <,001 | 0 |  |  |

GLP-1 = Glucagon-like peptide-1, ACEI= Angiotensin-converting enzyme inhibitors, ARB=Angiotensin receptor blockers,
