# Supplementary material for: Identifying high-risk combinations of metformin during COVID-19
Source: PLoS One. 2026 Mar 4;21(3):e0343979. doi: 10.1371/journal.pone.0343979 (PMC12959685; doi:10.1371/journal.pone.0343979)
Supplement: S9 Table — (DOCX) [file pone.0343979.s008.docx]

S9 Table Logistic regression for metformin+sulfonylurea vs metformin only after weighing

|  | B | S.E. | Wald | df | Sig. | Exp(B) | 95% C.I.for EXP(B) | |
| --- | --- | --- | --- | --- | --- | --- | --- | --- |
|  |  |  |  |  |  |  | Lower | Upper |
| Age | 0.061 | 0.004 | 300.477 | 1 | <,001 | 1.063 | 1.056 | 1.071 |
| Diabetes duration shorter than 7 years | -0.191 | 0.068 | 7.79 | 1 | 0.005 | 0.826 | 0.722 | 0.945 |
| Sex (female) | -0.85 | 0.069 | 151.376 | 1 | <,001 | 0.428 | 0.373 | 0.49 |
| ACEI | -0.957 | 0.15 | 40.98 | 1 | <,001 | 0.384 | 0.286 | 0.515 |
| ARB | -1.523 | 0.178 | 73.108 | 1 | <,001 | 0.218 | 0.154 | 0.309 |
| Vaccination p1 | -2.34 | 0.362 | 41.803 | 1 | <,001 | 0.096 | 0.047 | 0.196 |
| Vaccination p2 | -0.114 | 0.07 | 2.657 | 1 | 0.103 | 0.892 | 0.778 | 1.023 |
| Vaccination b | -0.269 | 0.199 | 1.824 | 1 | 0.177 | 0.764 | 0.517 | 1.129 |
| Neoplasm | 0.105 | 0.102 | 1.062 | 1 | 0.303 | 1.111 | 0.91 | 1.356 |
| Arterial hypertension | 0.224 | 0.101 | 4.89 | 1 | 0.027 | 1.251 | 1.026 | 1.526 |
| Ishemic heart disease | -0.022 | 0.097 | 0.05 | 1 | 0.823 | 0.979 | 0.809 | 1.183 |
| Cardiomyopathy | -0.051 | 0.113 | 0.201 | 1 | 0.654 | 0.951 | 0.762 | 1.186 |
| Cerebrovscular diseases | -0.041 | 0.116 | 0.126 | 1 | 0.723 | 0.96 | 0.765 | 1.204 |
| Circulatory diseases except hypertension | 0.316 | 0.084 | 14.168 | 1 | <,001 | 1.372 | 1.163 | 1.617 |
| Chronic lower respiratory diseases | 0.268 | 0.145 | 3.429 | 1 | 0.064 | 1.308 | 0.984 | 1.737 |
| Other chronic obstructive lung diseases | 0.153 | 0.175 | 0.767 | 1 | 0.381 | 1.166 | 0.827 | 1.642 |
| Chronic kidney disease | 0.527 | 0.171 | 9.442 | 1 | 0.002 | 1.694 | 1.21 | 2.37 |
| Metformin+sulfonylurea_vs_metformin only | 0.178 | 0.083 | 4.589 | 1 | 0.032 | 1.195 | 1.015 | 1.406 |
| Constant | -7.707 | 0.274 | 793.522 | 1 | <,001 | 0 |  |  |

ACEI= Angiotensin-converting enzyme inhibitors, ARB=Angiotensin receptor blockers
