# Supplementary material for: Identifying high-risk combinations of metformin during COVID-19
Source: PLoS One. 2026 Mar 4;21(3):e0343979. doi: 10.1371/journal.pone.0343979 (PMC12959685; doi:10.1371/journal.pone.0343979)
Supplement: S11 Table — (DOCX) [file pone.0343979.s010.docx]

S10 Table Logistic regression for metformin+SGLT-2 inhibitor vs metformin only prior weighing

Nagelkerke R Square 23.1%

Sig. <0.001

|  | B | S.E. | Wald | df | Sig. | Exp(B) | 95% C.I.for EXP(B) | |
| --- | --- | --- | --- | --- | --- | --- | --- | --- |
|  |  |  |  |  |  |  | Lower | Upper |
| Age | 0.067 | 0.004 | 290.931 | 1 | <,001 | 1.07 | 1.061 | 1.078 |
| Diabetes duration shorter than 7 years | -0.285 | 0.121 | 5.571 | 1 | 0.018 | 0.752 | 0.594 | 0.953 |
| Sex (female) | -0.87 | 0.08 | 117.237 | 1 | <,001 | 0.419 | 0.358 | 0.491 |
| ACEI | -0.094 | 0.081 | 1.339 | 1 | 0.247 | 0.911 | 0.777 | 1.067 |
| ARB | -0.351 | 0.242 | 2.094 | 1 | 0.148 | 0.704 | 0.438 | 1.132 |
| Vaccination p1 | -1.013 | 0.173 | 34.213 | 1 | <,001 | 0.363 | 0.259 | 0.51 |
| Vaccination p2 | -1.602 | 0.208 | 59.61 | 1 | <,001 | 0.201 | 0.134 | 0.303 |
| Vaccination b1 | -2.227 | 0.397 | 31.449 | 1 | <,001 | 0.108 | 0.049 | 0.235 |
| Neoplasm | 0.222 | 0.114 | 3.787 | 1 | 0.052 | 1.249 | 0.998 | 1.561 |
| Arterial hypertension | 0.221 | 0.116 | 3.638 | 1 | 0.056 | 1.247 | 0.994 | 1.565 |
| Ishemic heart disease | -0.062 | 0.113 | 0.304 | 1 | 0.581 | 0.94 | 0.753 | 1.173 |
| Cardiomyopathy | -0.041 | 0.133 | 0.093 | 1 | 0.761 | 0.96 | 0.739 | 1.247 |
| Cerebrovscular diseases | -0.021 | 0.137 | 0.023 | 1 | 0.881 | 0.98 | 0.749 | 1.281 |
| Circulatory diseases except hypertension | 0.268 | 0.097 | 7.601 | 1 | 0.006 | 1.307 | 1.08 | 1.581 |
| Chronic lower respiratory diseases | 0.171 | 0.172 | 0.978 | 1 | 0.323 | 1.186 | 0.846 | 1.663 |
| Other chronic obstructive lung diseases | 0.31 | 0.205 | 2.3 | 1 | 0.129 | 1.364 | 0.913 | 2.036 |
| Chronic kidney disease | 0.553 | 0.2 | 7.646 | 1 | 0.006 | 1.738 | 1.175 | 2.572 |
| Metformin+SGLT-2 inhibitor_vs_metformin only | 0.173 | 0.186 | 0.871 | 1 | 0.351 | 1.189 | 0.827 | 1.71 |
| Constant | -7.98 | 0.316 | 635.747 | 1 | <,001 | 0 |  |  |

SGLT-2 = Sodium-glucose co-transporter 2, ACEI= Angiotensin-converting enzyme inhibitors, ARB=Angiotensin receptor blockers
