# Supplementary material for: Identifying high-risk combinations of metformin during COVID-19
Source: PLoS One. 2026 Mar 4;21(3):e0343979. doi: 10.1371/journal.pone.0343979 (PMC12959685; doi:10.1371/journal.pone.0343979)
Supplement: S12 Table — (DOCX) [file pone.0343979.s011.docx]

S11 Table Logistic regression for metformin+SGLT-2 inhibitor vs metformin only after weighing

|  | B | S.E. | Wald | df | Sig. | Exp(B) | 95% C.I.for EXP(B) | |
| --- | --- | --- | --- | --- | --- | --- | --- | --- |
|  |  |  |  |  |  |  | Lower | Upper |
| Age | 0.065 | 0.004 | 262.832 | 1 | <,001 | 1.068 | 1.059 | 1.076 |
| Diabetes duration shorter than 7 years | -0.252 | 0.08 | 9.85 | 1 | 0.002 | 0.777 | 0.664 | 0.91 |
| Sex (female) | -0.896 | 0.081 | 122.958 | 1 | <,001 | 0.408 | 0.348 | 0.478 |
| ACEI | -0.106 | 0.081 | 1.685 | 1 | 0.194 | 0.9 | 0.767 | 1.055 |
| ARB | -0.408 | 0.248 | 2.703 | 1 | 0.1 | 0.665 | 0.409 | 1.082 |
| Vaccination p1 | -1.001 | 0.173 | 33.616 | 1 | <,001 | 0.367 | 0.262 | 0.515 |
| Vaccination p2 | -1.606 | 0.207 | 59.902 | 1 | <,001 | 0.201 | 0.134 | 0.301 |
| Vaccination b1 | -2.246 | 0.402 | 31.138 | 1 | <,001 | 0.106 | 0.048 | 0.233 |
| Neoplasm | 0.207 | 0.115 | 3.201 | 1 | 0.074 | 1.229 | 0.98 | 1.542 |
| Arterial hypertension | 0.226 | 0.117 | 3.743 | 1 | 0.053 | 1.253 | 0.997 | 1.575 |
| Ishemic heart disease | -0.036 | 0.113 | 0.098 | 1 | 0.754 | 0.965 | 0.773 | 1.205 |
| Cardiomyopathy | 0.002 | 0.134 | 0 | 1 | 0.986 | 1.002 | 0.772 | 1.302 |
| Cerebrovscular diseases | -0.019 | 0.138 | 0.019 | 1 | 0.892 | 0.981 | 0.748 | 1.287 |
| Circulatory diseases except hypertension | 0.24 | 0.098 | 5.967 | 1 | 0.015 | 1.271 | 1.049 | 1.541 |
| Chronic lower respiratory diseases | 0.185 | 0.174 | 1.134 | 1 | 0.287 | 1.204 | 0.856 | 1.692 |
| Other chronic obstructive lung diseases | 0.315 | 0.206 | 2.33 | 1 | 0.127 | 1.37 | 0.915 | 2.052 |
| Chronic kidney disease | 0.529 | 0.201 | 6.902 | 1 | 0.009 | 1.697 | 1.144 | 2.519 |
| Metformin+SGLT-2 inhibitor_vs_metformin only | 0.076 | 0.169 | 0.201 | 1 | 0.654 | 1.079 | 0.774 | 1.504 |
| Constant | -7.923 | 0.309 | 657.726 | 1 | <,001 | 0 |  |  |

SGLT-2 = Sodium-glucose co-transporter 2, ACEI= Angiotensin-converting enzyme inhibitors, ARB=Angiotensin receptor blockers
