# Supplementary material for: Identifying high-risk combinations of metformin during COVID-19
Source: PLoS One. 2026 Mar 4;21(3):e0343979. doi: 10.1371/journal.pone.0343979 (PMC12959685; doi:10.1371/journal.pone.0343979)
Supplement: S13 Table — (DOCX) [file pone.0343979.s012.docx]

S12 Table Logistic regression for metformin+pioglitazone vs metformin only prior weighing

Nagelkerke R Square 0.231

Sig. <0.001

|  | B | S.E. | Wald | df | Sig. | Exp(B) | 95% C.I.for EXP(B) | |
| --- | --- | --- | --- | --- | --- | --- | --- | --- |
|  |  |  |  |  |  |  | Lower | Upper |
| Age | 0.065 | 0.004 | 251.442 | 1 | <,001 | 1.067 | 1.058 | 1.075 |
| Diabetes duration shorter than 7 years | -0.219 | 0.081 | 7.327 | 1 | 0.007 | 0.803 | 0.685 | 0.941 |
| Sex (female) | -0.861 | 0.081 | 113.271 | 1 | <,001 | 0.423 | 0.361 | 0.496 |
| ACEI | -0.079 | 0.082 | 0.934 | 1 | 0.334 | 0.924 | 0.787 | 1.085 |
| ARB | -0.377 | 0.249 | 2.302 | 1 | 0.129 | 0.686 | 0.421 | 1.116 |
| Vaccination p1 | -1.03 | 0.178 | 33.574 | 1 | <,001 | 0.357 | 0.252 | 0.506 |
| Vaccination p2 | -1.568 | 0.212 | 54.524 | 1 | <,001 | 0.208 | 0.137 | 0.316 |
| Vaccination b1 | -2.337 | 0.427 | 29.989 | 1 | <,001 | 0.097 | 0.042 | 0.223 |
| Neoplasm | 0.208 | 0.116 | 3.223 | 1 | 0.073 | 1.231 | 0.981 | 1.544 |
| Arterial hypertension | 0.226 | 0.119 | 3.638 | 1 | 0.056 | 1.254 | 0.994 | 1.582 |
| Ishemic heart disease | -0.076 | 0.116 | 0.429 | 1 | 0.513 | 0.927 | 0.739 | 1.163 |
| Cardiomyopathy | -0.021 | 0.135 | 0.025 | 1 | 0.875 | 0.979 | 0.752 | 1.274 |
| Cerebrovscular diseases | -0.011 | 0.137 | 0.007 | 1 | 0.935 | 0.989 | 0.756 | 1.294 |
| Circulatory diseases except hypertension | 0.28 | 0.098 | 8.152 | 1 | 0.004 | 1.323 | 1.092 | 1.604 |
| Chronic lower respiratory diseases | 0.211 | 0.173 | 1.495 | 1 | 0.221 | 1.235 | 0.88 | 1.732 |
| Other chronic obstructive lung diseases | 0.279 | 0.205 | 1.854 | 1 | 0.173 | 1.322 | 0.885 | 1.976 |
| Chronic kidney disease | 0.554 | 0.203 | 7.443 | 1 | 0.006 | 1.741 | 1.169 | 2.592 |
| Metformin+pioglitazone_vs_metformin only | -0.074 | 0.267 | 0.076 | 1 | 0.783 | 0.929 | 0.55 | 1.568 |
| Constant | -7.923 | 0.314 | 637.375 | 1 | <,001 | 0 |  |  |

ACEI= Angiotensin-converting enzyme inhibitors, ARB=Angiotensin receptor blockers
