# Supplementary material for: Identifying high-risk combinations of metformin during COVID-19
Source: PLoS One. 2026 Mar 4;21(3):e0343979. doi: 10.1371/journal.pone.0343979 (PMC12959685; doi:10.1371/journal.pone.0343979)
Supplement: S14 Table — (DOCX) [file pone.0343979.s013.docx]

S13 Table Logistic regression for metformin+pioglitazone vs metformin only after weighing

|  | B | S.E. | Wald | df | Sig. | Exp(B) | 95% C.I.for EXP(B) | |
| --- | --- | --- | --- | --- | --- | --- | --- | --- |
|  |  |  |  |  |  |  | Lower | Upper |
| Age | 0.064 | 0.004 | 249.571 | 1 | <,001 | 1.066 | 1.058 | 1.075 |
| Diabetes duration shorter than 7 years | -0.224 | 0.081 | 7.633 | 1 | 0.006 | 0.799 | 0.682 | 0.937 |
| Sex (female) | -0.857 | 0.081 | 111.788 | 1 | <,001 | 0.424 | 0.362 | 0.497 |
| ACEI | -0.083 | 0.082 | 1.029 | 1 | 0.31 | 0.92 | 0.783 | 1.081 |
| ARB | -0.375 | 0.249 | 2.279 | 1 | 0.131 | 0.687 | 0.422 | 1.118 |
| Vaccination p1 | -1.035 | 0.178 | 33.688 | 1 | <,001 | 0.355 | 0.251 | 0.504 |
| Vaccination p2 | -1.567 | 0.213 | 54.139 | 1 | <,001 | 0.209 | 0.137 | 0.317 |
| Vaccination b1 | -2.334 | 0.428 | 29.758 | 1 | <,001 | 0.097 | 0.042 | 0.224 |
| Neoplasm | 0.205 | 0.116 | 3.117 | 1 | 0.077 | 1.228 | 0.978 | 1.542 |
| Arterial hypertension | 0.217 | 0.118 | 3.36 | 1 | 0.067 | 1.243 | 0.985 | 1.567 |
| Ishemic heart disease | -0.078 | 0.116 | 0.452 | 1 | 0.502 | 0.925 | 0.736 | 1.162 |
| Cardiomyopathy | -0.023 | 0.135 | 0.029 | 1 | 0.866 | 0.977 | 0.75 | 1.274 |
| Cerebrovscular diseases | -0.023 | 0.138 | 0.026 | 1 | 0.871 | 0.978 | 0.745 | 1.282 |
| Circulatory diseases except hypertension | 0.275 | 0.098 | 7.84 | 1 | 0.005 | 1.317 | 1.086 | 1.596 |
| Chronic lower respiratory diseases | 0.215 | 0.173 | 1.538 | 1 | 0.215 | 1.239 | 0.883 | 1.74 |
| Other chronic obstructive lung diseases | 0.276 | 0.206 | 1.801 | 1 | 0.18 | 1.318 | 0.881 | 1.972 |
| Chronic kidney disease | 0.56 | 0.204 | 7.551 | 1 | 0.006 | 1.75 | 1.174 | 2.609 |
| Metformin+pioglitazone_vs_metformin only | -0.198 | 0.262 | 0.569 | 1 | 0.451 | 0.821 | 0.491 | 1.372 |
| Constant | -7.879 | 0.312 | 635.911 | 1 | <,001 | 0 |  |  |

ACEI= Angiotensin-converting enzyme inhibitors, ARB=Angiotensin receptor blockers
