# Supplementary material for: Identifying high-risk combinations of metformin during COVID-19
Source: PLoS One. 2026 Mar 4;21(3):e0343979. doi: 10.1371/journal.pone.0343979 (PMC12959685; doi:10.1371/journal.pone.0343979)
Supplement: S15 Table — (DOCX) [file pone.0343979.s014.docx]

S14 Table Logistic regression for metformin+GLP-1 agonist vs metformin only prior weighing

Nagelkerke R Square 0.231

Sig. <0.001

|  | B | S.E. | Wald | df | Sig. | Exp(B) | 95% C.I.for EXP(B) | |
| --- | --- | --- | --- | --- | --- | --- | --- | --- |
|  |  |  |  |  |  |  | Lower | Upper |
| Age | 0.067 | 0.004 | 282.669 | 1 | <,001 | 1.069 | 1.061 | 1.078 |
| Diabetes duration shorter than 7 years | -0.255 | 0.122 | 4.372 | 1 | 0.037 | 0.775 | 0.61 | 0.984 |
| Sex (female) | -0.858 | 0.08 | 114.078 | 1 | <,001 | 0.424 | 0.362 | 0.496 |
| ACEI | -0.073 | 0.081 | 0.802 | 1 | 0.37 | 0.93 | 0.793 | 1.091 |
| ARB | -0.297 | 0.237 | 1.571 | 1 | 0.21 | 0.743 | 0.467 | 1.182 |
| Vaccination p1 | -0.974 | 0.173 | 31.565 | 1 | <,001 | 0.378 | 0.269 | 0.53 |
| Vaccination p2 | -1.604 | 0.207 | 59.865 | 1 | <,001 | 0.201 | 0.134 | 0.302 |
| Vaccination b1 | -2.394 | 0.426 | 31.619 | 1 | <,001 | 0.091 | 0.04 | 0.21 |
| Neoplasm | 0.197 | 0.116 | 2.904 | 1 | 0.088 | 1.218 | 0.971 | 1.528 |
| Arterial hypertension | 0.273 | 0.12 | 5.196 | 1 | 0.023 | 1.314 | 1.039 | 1.663 |
| Ishemic heart disease | -0.1 | 0.115 | 0.757 | 1 | 0.384 | 0.905 | 0.723 | 1.133 |
| Cardiomyopathy | -0.023 | 0.133 | 0.029 | 1 | 0.865 | 0.978 | 0.753 | 1.269 |
| Cerebrovscular diseases | -0.037 | 0.137 | 0.071 | 1 | 0.79 | 0.964 | 0.736 | 1.262 |
| Circulatory diseases except hypertension | 0.285 | 0.097 | 8.581 | 1 | 0.003 | 1.329 | 1.099 | 1.608 |
| Chronic lower respiratory diseases | 0.203 | 0.171 | 1.415 | 1 | 0.234 | 1.225 | 0.877 | 1.711 |
| Other chronic obstructive lung diseases | 0.308 | 0.202 | 2.323 | 1 | 0.127 | 1.36 | 0.916 | 2.02 |
| Chronic kidney disease | 0.591 | 0.198 | 8.945 | 1 | 0.003 | 1.806 | 1.226 | 2.66 |
| Metformin+GLP-1_vs_metformin only | 0.872 | 0.229 | 14.509 | 1 | <,001 | 2.391 | 1.527 | 3.745 |
| Constant | -8.051 | 0.321 | 628.169 | 1 | <,001 | 0 |  |  |

GLP-1 = Glucagon-like peptide-1, ACEI= Angiotensin-converting enzyme inhibitors, ARB=Angiotensin receptor blockers,
